# Clinical application of chromosomal microarray analysis for fetuses with craniofacial malformations

**DOI:** 10.1186/s13039-020-00502-5

**Published:** 2020-08-25

**Authors:** Chenyang Xu, Yanbao Xiang, Xueqin Xu, Lili Zhou, Huanzheng Li, Xueqin Dong, Shaohua Tang

**Affiliations:** 1 Center of Prenatal Diagnosis, Wenzhou Central Hospital, Wenzhou, China; 2 Key Laboratory of Medical Genetic, School of Laboratory Medicine and Life Science, Wenzhou Medical University, Wenzhou, China

**Keywords:** Chromosomal microarray analysis, Craniofacial malformation, Prenatal diagnosis

## Abstract

**Background:**

The potential correlations between chromosomal abnormalities and craniofacial malformations (CFMs) remain a challenge in prenatal diagnosis. This study aimed to evaluate 118 fetuses with CFMs by applying chromosomal microarray analysis (CMA) and G-banded chromosome analysis.

**Results:**

Of the 118 cases in this study, 39.8% were isolated CFMs (47/118) whereas 60.2% were non-isolated CFMs (71/118). The detection rate of chromosomal abnormalities in non-isolated CFM fetuses was significantly higher than that in isolated CFM fetuses (26/71 vs. 7/47, *p* = 0.01). Compared to the 16 fetuses (16/104; 15.4%) with pathogenic chromosomal abnormalities detected by karyotype analysis, CMA identified a total of 33 fetuses (33/118; 28.0%) with clinically significant findings. These 33 fetuses included cases with aneuploidy abnormalities (14/118; 11.9%), microdeletion/microduplication syndromes (9/118; 7.6%), and other pathogenic copy number variations (CNVs) only (10/118; 8.5%).We further explored the CNV/phenotype correlation and found a series of clear or suspected dosage-sensitive CFM genes including *TBX1*, *MAPK1*, *PCYT1A*, *DLG1*, *LHX1*, *SHH*, *SF3B4, FOXC1*, *ZIC2*, *CREBBP*, *SNRPB*, and *CSNK2A1*.

**Conclusion:**

These findings enrich our understanding of the potential causative CNVs and genes in CFMs. Identification of the genetic basis of CFMs contributes to our understanding of their pathogenesis and allows detailed genetic counselling.

## Background

Craniofacial malformations (CFMs) are among the most common congenital birth malformations in humans, with orofacial clefts accounting for approximately 13% of congenital malformations in all live births [1]. The majority of CFMs occur in fetuses without a family history. Thus, it is particularly important to evaluate the integrity of the craniofacial structures of fetuses using ultrasonographic screening. Generally, fetal CFMs include defects such as cranial malformations, ocular malformations, nasal dysplasia, and orofacial defects, among others. These defects may be isolated variations or may occur in combination with other congenital structural abnormalities such as central nervous system abnormalities, cardiac defects, abdominal wall defects, skeletal defects, and so on. Due to the similar phenotypes of craniofacial syndromes with and without multiple organ involvement, prenatal genetic counseling can be challenging. Although the causes of CFMs are currently unclear, genetic analysis can help to provide a genetic basis for prenatal diagnosis and can also contribute to our understanding of the pathogenesis of CFMs.

Conventional karyotyping is the classic method for detecting aneuploidy or chromosomal rearrangements [2, 3]. However, this approach has gradually been replaced by chromosomal microarray analysis (CMA) due to its low resolution and low detection efficiency. Recently, CMA has been recommended for prenatal diagnosis when fetal abnormalities are detected by ultrasound [4]. Copy number variations (CNVs) in simple cranial or facial malformations have also been reported [5, 6]; however, the comprehensive CMA assessment of fetuses with CFMs is limited. In the present study, we reviewed the clinical and molecular findings of 118 fetuses with CFMs to explore the clinical significance of CNVs in each case. This study aimed to provide useful information for prenatal diagnosis of CFMs and related genetic counseling.

## Results

### Fetal ultrasound findings

As shown in Table 1, CFMs, including cranial malformations (41, 34.7%), orofacial clefts (44, 37.3%), ocular and orbital malformations (6, 5.1%), nasal deformities (5, 4.2%), ear abnormalities (3, 2.5%), macroglossia (1, 0.8%), micrognathia (1, 0.8%), and complex CFMs (17, 14.4%) were observed in 118 fetuses. Complex CFMs refer to abnormalities involving two or more different cranial or facial features. Of these 118 cases, 47 (39.8%) were isolated CFMs while 71 (60.2%) were non-isolated CFMs.

**Table 1:** Phenotypic characteristics of 118 fetuses with CFMs

Abnormalities	Isolated CFM (N)	Non-isolated CFM (N)	Referred cases (N)
*Cranial malformations*			*41 (34.7%)*
Microcephaly	5	4	9
Macrocephaly	1	5	6
Defect in the skull bone	0	13	13
Abnormal skull shape	3	10	13
*Orofacial clefts*			*44 (37.3%)*
cleft lip	6	4	10
cleft palate	0	1	1
cleft lip and palate	19	14	33
*Ocular and orbital malformations*			*6 (5.1%)*
Hypertelorism	0	3	3
Hypotelorism	0	2	2
Microphthalmia, Cataract	0	1	1
*Nasal deformity*	5	0	*5 (4.2%)*
*Ear abnormality*	2	1	*3 (2.5%)*
*Macroglossia*	0	1	*1 (0.8%)*
*Micrognathia*	1	0	*1(0.8%)*
*Complex CFMs*	5	12	*17 (14.4%)*
Total	47 (39.8%)	71 (60.2%)	118 (100%)

*CFM* craniofacial malformation

### Conventional G-banded cytogenetic analysis findings

Samples for karyotype analysis were obtained from 104 fetuses (40 samples from amniocentesis and 64 samples from cordocentesis); another 14 samples obtained from aborted fetuses were excluded. Successful karyotyping results indicated that 16 (16/104; 15.4%) fetuses had chromosomal abnormalities; 11 cases showed aneuploidy including trisomy 13 (*n* = 5), trisomy 18 (*n* = 4), trisomy 21 (*n* = 1), and mosaicism 45,X[32]/46,XY[3] (*n* = 1). Another 5 cases showed chromosomal structural aberrations: 46,XN,der(13)t(4;13)(q35;q31), 46,XN,der(13)t(13;16)(q32;q23), 46,XN,del(7)(q34), 46,XN,rec(6)dup(6q)inv(6)(p25q22), and 45,XN,der(14)t(14;20)(p13;p11.2),-20[17]/46,XN[17].

### CMA findings

An interpretable CMA profile was obtained for all 118 tested genomic DNA samples. Clinically significant results were found in 33 cases (33/118; 28.0%), including 14 cases (14/118; 11.9%) with chromosomal aneuploidies and 19 cases (19/118; 16.1%) with Pathogenic (P) or Likely Pathogenic (LP) CNVs.

**Fig. 1:**
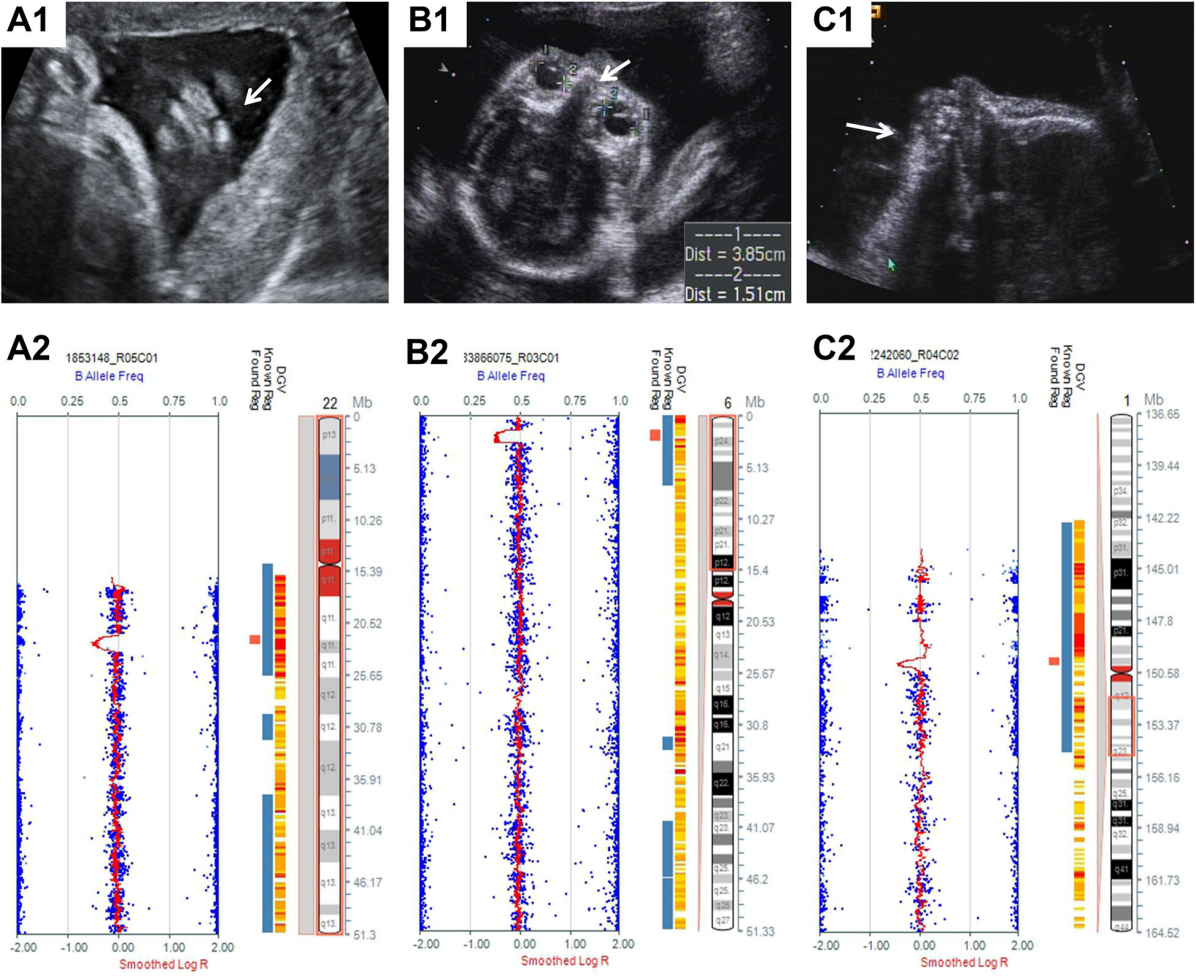
Abnormal ultrasonographic findings and the identified pCNVs of three selected fetuses. **a** A 0.96 Mb deletion related to 22q11.2 distal deletion syndrome (A2) was identified in fetus 17 with cleft lip (A1). **b** A 1.20 Mb deletion of 6p25.3p25.2 (B2) was identified in fetus 32 with hypertelorism (B1). **c** A 0.44 Mb deletion of 1q21.11q21.2 (C2) was identified in fetus 33 with micrognathia (C1)

In the 14 cases with CMA results indicating chromosomal aneuploidies, 6 fetuses had trisomy 13, 5 fetuses had trisomy 18, one fetus had trisomy 21, one fetus had monosomy X, and one fetus had mosaic copy gain of the X chromosome in approximately 20% of cells (Table 2). Of the 19 cases with P/LP CNVs, we identified 9 fetuses (9/118; 7.6%) with CNVs related to known microdeletion or microduplication syndromes (MMSs). These included 22q11 deletion syndrome (*n* = 3), 22q11 duplication syndrome (*n* = 1), 7q11.23 duplication syndrome (*n* = 1), 3q29 microdeletion syndrome (*n* = 1), 16p11.2 microduplication syndrome (*n* = 1), renal cysts and diabetes syndrome (*n* = 1), and 8p23.1 duplication syndrome (*n* = 1). In addition to MMSs, we identified a further 20 pathogenic CNVs from 11 fetuses. These CNVs involved deletions of 1q21, 4q32q35, 6p25p25, 7q34q36, 11q24q25, 13q31q34, 20p13p11, and Xq26q28, and duplications of 4q32q35, 4q35, 6q22q25, 6q25q27, 7p22p21, 8p23p23, 8p23, 16q23q24, and 16p13p13 (Table 3 and Fig. 1). Among these cases, fetus 23 was found to have 8p23.1 duplication syndrome combined with another 4 pathogenic CNVs.

**Table 2:** CFMs fetuses with chromosomal aneuploidy abnormalities identified by CMA and karyotype analysis

Case	Karyotype	CMA results	Craniofacial malformations	Other malformations
1	47,XN,+13	arr(13)×3	CLP	Gallbladder enlargement; ES; Hyperechogenic kidneys; Strephenopodia; Small stomach bubble
2	47,XN,+13	arr(13)×3	Microphthalmia, CLP	Gallbladder enlargement; DW; Hyperechogenic kidneys
3	47,XN,+13	arr(13)×3	Abnormal skull shape, CLP	HPE, DK, TOF
4	47,XN,+13	arr(13)×3	Lemon-shaped skull	Bilateral cerebral ventriculomegaly; SB
5	47,XN,+13	arr(13)×3	CLP	–
6	NA	arr(13)×3	Skull defect	Encephalocele
7	47,XN,+18	arr(18)×3	Abnormal skull shape	HPE; absent radius; VSD; SUA
8	47,XN,+18	arr(18)×3	Microtia, Abnormal pinna	CHD, CH, abnormal hand posture, SUA, polyhydramnios
9	NA	arr(18)×3	Midface depression	Limb body wall complex
10	47,XN,+18	arr(18)×3	CLP	CPC; VSD
11	47,XN,+18	arr(18)×3	Strawberry-shaped skull	Overlapping hands, CPC, LPCM, SUA
12	47,XN,+21	arr(21)×3	Abnormal skull shape	–
13	45,X[32]/46,XY[3]	arr(X)×1	CL	–
14	NA	arr(X)×1~2, (Y)×1	Skull defect	Anencephaly; Enlarged bladder

*CFM* craniofacial malformation, *CH* cerebral hernia, *CHD* complex congenital heart disease, *CLP* cleft lip and palate, *CL* cleft lip, *CMA* chromosomal microarray analysis, *CPC* choroid plexus cysts, *DK* duplex kidney, *DWM* Dandy-Walker malformation, *ES* esophageal stenosis, *HPE* Holoprosencephaly, *LPCM* low placed conus medullaris, *SB* Spina bifida, *SUA* single umbilical artery, *TOF* tetralogy of Fallot, *VSD* ventricular septal defect; XN, XX or XY

**Table 3:** CFMs fetuses with microdeletion/microduplication syndromes and other pCNVs

Case	Craniofacial malformations	Other findings	Karyotype	Candidate Gene	Clinical significance	CMA results	Size(Mb)	Inheritance
15	CLP	–	46,XN	*TBX1*	P (22q11 proximal deletion syndrome)	22q11.21(18895227_21460220)×1	2.56	NA
16	CLP	CHD	46,XN	*TBX1*	P (22q11 proximal deletion syndrome)	22q11.21(18895227_21445064)×1	2.55	De novo
17	CL	RVE, RAE, APVD	46,XN	*MAKP1*	P (22q11.2 distal deletion syndrome)	22q11.2(21798907_22762651)×1	0.96	De novo
18	CLP	–	NA	*TBX1*	P (22q11 duplication syndrome)	22q11.2(18648855_21800471)×3	3.15	Maternal
19	Skull defect	Anencephaly	NA	*_*	P (7q11.23 duplication syndrome)	7q11.23(72722981_74494207)×3	1.77	NA
					VOUS	14q12(25333115_26945366)×3	1.61	NA
20	CLP	–	46,XN	*PCYT1A, DLG1*	P (3q29 microdeletion syndrome)	3q29(195678474_197340833)×1	1.66	NA
21	Macrocephaly	Hydronephrosis	46,XN	*LHX1*	P (Rrenal cysts and diabetes syndrome)	17q12(34462281_36249565)×1	1.78	De novo
22	CLP	IUGR	46,XN	*_*	VOUS	14q32.33(104871320_106251148)×1	1.38	Maternal
					LP (16p11.2 microduplication syndrome)	16p11.2(29681582_30190029)×3	0.51	De novo
23	Abnormal skull shape	Absent gallbladder, RD, PE, IUGR, HPE	NA	*ZIC2*	P	4q32.2q35.2(163980423_190880409)×3	26.89	De novo
					P	8p23.3p23.1(176818_6974050)×3	6.80	De novo
					P(8p23.1 duplication syndrome)	8p23.1(8101641_11394233)×3	3.29	De novo
					P	13q31.3q34(92884370_115106996)×1	22.22	De novo
					P	Xq26.2q28(130488944_154929412)×1	24.40	De novo
24	Abnormal skull shape	HPE	46,XN,der(13)t(4;13)(q35;q31)	*ZIC2*	P	4q35.1q35.2(183907715_190880409)×3	6.97	Paternal balanced translocation
					P	13q31.3q34(94514343_115106996)×1	20.59	
25	CLP	VWT	46,XN,der(13)t(13;16)(q32;q23)	*ZIC2*	P	13q32.1q34(96311577_115106996)×1	18.8	Maternal balanced translocation
					P	16q23.2q24.3(81148438_90148796)×3	9.00	
26	CL	DWM	46,XN	*SHH*	P	7p22.3p21.2(43376_15044564)×3	15.00	NA
					P	7q34q36.3(142326472_159119707)×1	16.79	
27	Abnormal skull shape, Hypotelorism, beaked nose	HPE, absent radius	46,XN,del(7)(q34)	*SHH*	P	7q34q36.3(138831707_159119486)×1	20.28	De novo
28	Midfacial hypoplasia	BPC, Hyperechogenic kidneys, LM, LPCM	46,XN,rec(6)dup(6q)inv(6)(p25q22)	*FOXC1*	P	6p25.3p25.1(156974_5395099)×1	5.24	Maternal inversion
					P	6q22.32q25.3(126253838_170914297)×3	44.66	
29	Microcephaly	RAA, Persistent LSVC	45,XN,der(14)t(14;20) (p13; p11.2),-20 [17]/46,XN[17]	*SNRPB, CSNK2A1*	P	20p13p11.21(61661_21268329)×1[0.4]	21.21	De novo
30	CP	PFL, NT thickening, Omphalocele	NA	*HPGD* (AR)	P	4q32.3q35.2(169998230_190880409)×1	20.88	Maternal balanced translocation
					P	6q25.3q27(158387117_170898549)×3	12.51	
31	Midfacial hypoplasia, flat nose, prominent maxilla	MCDK, VSD, Persistent LSVC, SUA	46,XN	*CREBBP*	P	11q24.1q25(122446233_134944006)×1	12.49	De novo
					P	16p13.3p13.12(105320_12986742)×3	12.88	De novo
32	Hypertelorism	ICL, Arachnoid cyst	46,XN	*FOXC1*	P	6p25.3p25.2(1482077_2681511)×1	1.20	De novo
33	Micrognathia	–	46,XN	*SF3B4*	P	1q21.2(149815079_150260948)×1	0.44	De novo

*APVD* anomalous pulmonary venous drainage, *BPC* blake’s pouch cyst, *CFM* craniofacial malformation, *CHD* complex heart disease, *CL* cleft lip, *CLP* cleft lip and palate, *CMA* chromosomal microarray analysis, *CNV* copy number variant, *CP* cleft palate, *DWM* Dandy-Walker malformation, *HPE* Holoprosencephaly, *ICL* intracranial cystic lesions, *IUGR* intrauterine growth retardation, *LM* Limb Malformations, *LP* likely pathogenic, *LPCM* low placed conus medullaris, *LSVC* left superior vena cava, *MCDK* multicystic dysplastic kidney, *NA* not available, *NT* nuchal translucency, *P* pathogenic, *PE* pericardial effusion, *PFL* posterior Fossa Lesions, *RAE* right atrial enlargement, *RAA* right aortic arch, *RD* Renal dysplasia, *RVE* right ventricular enlargement, *SUA* single umbilical artery, *VOUS* variant of unknown significance, *VWT* ventricular wall thickening, *VSD* ventricular septal defect; XN, XX or XY

All 33 fetuses identified to have P/LP CNVs were terminated in pregnancy or died in utero. Additionally, we found that 9 (9/118; 7.6%) fetuses that had variant of unknown significance CNVs. The remaining 76 cases (76/118; 64.4%) had no CMA abnormalities or only likely benign/benign CNVs.

### Identification of CFM-associated CNVs and genes

We further analyzed the associations between CFMs and these CNVs and identified the potential candidate genes within these regions. We screened several dosage-sensitive or suspected dosage-sensitive genes, including genes *TBX1* (22q11.21), *MAPK1* (22q11.22), *PCYT1A* (3q29), and *DLG1* (3q29) related to cleft lip/palate, *LHX1* (17q12) related to macrocephaly, *SF3B4* (1q21.2) related to micrognathia, *FOXC1* (6p25.3) related to ocular hypertelorism and midfacial hypoplasia, *ZIC2* (13q32.3) related to cleft lip/palate and abnormal skull shape, *SHH* (7q36.3) related to multiple CFMs, *CREBBP* (16p13.3) related to complex CFMs, and *SNRPB* or *CSNK2A1* (20p13) related to microcephaly.

### Comparison of chromosomal abnormality detection rates

Overall, as shown in Table 4, the detection rate of chromosomal abnormalities in non-isolated CFM fetuses was significantly higher than in isolated CFM fetuses (26/71 vs. 7/47; *p* = 0.01). However, there was no significant difference in the chromosomal abnormality detection rate of complex CFMs compared to simple CFMs (6/17 vs. 27/101; *p* > 0.05).

**Table 4:** Detection rate of clinically significant chromosomal aberrations in fetuses with various CFMs

*Malformation*	Simple CFMs							Complex CFMs (n/N)	Referred cases (n/N, %)
	Cranial malformation (n/N)	Orofacial cleft (n/N)	Nasal deformity (n/N)	Ocular malformation (n/N)	Ear abnormality (n/N)	Macroglossia (n/N)	Micrognathia (n/N)		
Isolated CFMs	1/9	5/25	0/5	0/0	0/2	0/0	1/1	0/5	7/47, 14.9%
Non-isolated CFMs	10/32	8/19	0/0	1/6	1/1	0/1	0/0	6/12	26/71, 36.6%
Total	11/41	13/44	0/5	1/6	1/3	0/1	1/1	6/17	33/118, 28.0%

*CFM* craniofacial malformation

## Discussion

Craniofacial anomalies are common in postnatal cases, especially in patients with nervous system disorders. The detection rate of prenatal cranial abnormalities is unclear; however, there is a high incidence of cranial abnormalities in neonates [7]. Nicolaides et al. (1993) reported a 7% incidence of facial defects in fetal malformations [8], of which orofacial clefts were the most common, with a prevalence of approximately 1 case per 700 deliveries [9]. In this study, it was observed that cranial abnormalities and orofacial clefts were most common, accounting for 40.6 and 43.6% of cases, respectively. This finding is consistent with the above reports.

CMA offers obvious advantages in improving the detection rate and identifying the pathogenicity of CNVs. Recently, there have also been several successive research publications on prenatal diagnosis of fetal cleft lip/palate and cranial anomalies with the use of CMA [5, 6]. In our study, cytogenetic karyotyping revealed abnormal karyotypes in 15.4% of fetuses and the detection rate increased by 12.6% with CMA. The incidence of chromosomal aberrations and CNVs was significantly higher than the 4.6 and 6.3%, respectively, reported in a recent study of fetal structural abnormalities [10]. The reason for the discrepancies between previous reports [10, 11] and our study is that we did not merely focus on isolated CFMs or cases with simple CFMs.

In this study, we attempted to determine the potential correlation between CNVs and CFMs in fetuses using CMA. This study identified 9 cases (cases 15–23) with CNVs relating to known chromosomal MMSs, of which 22q11 deletion/duplication syndrome was the most common, with an overall prevalence of 3.4% (4/118; 2 proximal deletion, 1 proximal duplication, and 1 distal deletion). 22q11 proximal deletion, also known as DiGeorge syndrome or velocardiofacial syndrome, involves more than 30 Mendelian genes; potential genes such as *TBX1, COMT, UFD1L, GNB1L, TRXR2, MED15,* and *RANBP1* were researched to explore the phenotype/CNV correlation. Cleft palate is among the most common problems in patients with this microdeletion, while simple cleft lip is only occasionally found [12]. According to previous reports, *TBX1* is considered to be responsible for cleft lip/palate phenotypes in both 22q11 deletion and 22q11 duplication [13]. Fetus 17, with heart abnormalities and cleft lip, was found to carry 22q11 distal deletion syndrome. Heart problems are a usual finding, but cleft lip only, without cleft palate, has never been reported within the clinical spectrum of this syndrome. This suggests that simple cleft lip may need to be included in the phenotype spectrum of 22q11 distal deletion syndrome. Although researchers such as Spineli-Silva argue that the cause of CHDs and craniofacial anomalies in patients with distal 22q11 deletion may be haploinsufficient *MAPK1* expression [14], the underlying mechanisms are still largely unknown.

In addition, we identified five distinct CNVs associated with rare MMSs, including two microdeletion syndromes (3q29 and 17q12) and three microduplication syndromes (7q11.23, 8p23.1 and 16p11.2). These syndromes are associated with a range of mental and physical disabilities as well as craniofacial abnormalities. We screened several candidate genes located in these regions that are involved in craniofacial development, such as *PCYT1A* (3q29), [15], *DLG1* (3q29), [16]), and *LHX1* (17q12), [17, 18]. Although *TBX6* is considered to be a key gene resulting in several major phenotypes in 16p11.2 duplication, potential genes associated with orofacial cleft in this region still require further exploration. Additionally, there is no reported correlation between 7q11.23 duplication and skull defects resulting from anencephaly, but this fragment has been confirmed as a pathogenic CNV of central nervous system development.

Other rare CNVs detected in the present study are also believed to contribute to the pathogenesis of CFMs. *ZIC2* in 13q23.3 (cases 23–25) has been identified as a key gene associated with several major CFMs resulting from holoprosencephaly [19]. Deletion in the chromosome 7q34q36.3-encompassing gene *SHH* was identified in cases 26 and 27; *SHH* is involved in the organization and morphology of the developing embryo and is known to be a key gene in craniofacial abnormalities such as microcephaly, hypotelorism, midface hypoplasia, and cleft lip/palate [20]. In case 33 with isolated micrognathia, a 0.44 Mb deletion in region 1q21.1 was identified; haploinsufficiency of gene *SF3B4* in 1q21.1 has been confirmed to be associated with micrognathia [21]. Additionally, there is also evidence of the pathogenicity of haploinsufficient *FOXC1* expression. Heterozygous deletion of *FOXC1* in 6p25.3 (cases 28 and 32) can lead to Axenfeld-Rieger syndrome (6p25 deletion syndrome); ocular hypertelorism and flat midface are prevalent in affected postnatal cases [22].

Case 31, which exhibited a maxillary protrusion, midface hypoplasia, and a flat nose, had a 12.49 Mb deletion in chromosome 11q24.1q25 and a 12.88 Mb duplication in chromosome 16p13.3p13.12 involving the gene *CREBBP*. There are several literature reports suggesting that duplication of the 16p13.3 region containing the *CREBBP* gene results in distinct similar facial dysmorphism [23], but, to date, no cases with duplication only encompassing the *CREBBP* gene have been reported. Case 29 had microcephaly < 2 SD and had a 21.21 Mb mosaic deletion in chromosome 20p13p11.21. Among 141 protein coding genes within this deletion region, mutations only in *SNRPB* and *CSNK2A1* have been reported to be associated with autosomal dominant microcephaly [24, 25]. However, to date, there is no evidence supporting their pathogenicity in haploinsufficiency. In case 30, we could not identify a gene specifically associated with the observed cleft palate; we only identified an autosomal recessive gene, *HPGD*, associated with a high-arched palate and without dose-sensitive reports [26]. We suspect a single mutation on the other chromosome may explain the observed phenotype. In the future, the function of candidate genes within the identified CNVs should be further investigated.

## Conclusion

The current findings enrich our understanding of the potential causative CNVs and genes in CFMs. We detected several CNVs, including nine MMS regions associated with CFMs, and found a series of clear or suspected dosage-sensitive CFM genes including *TBX1*, *MAPK1*, *PCYT1A*, *DLG1*, *LHX1*, *SHH*, *SF3B4, FOXC1*, *ZIC2*, *CREBBP*, *SNRPB*, and *CSNK2A1*.

## Methods

### Study subjects

The present study was approved by the institutional research ethics committee of our unit. All parents agreed to participate in the study and provided written informed consent. We retrospectively analyzed a cohort of 118 fetuses with CFMs that were diagnosed at the Wenzhou Prenatal Diagnosis Center between 2012 and 2019. All the pregnant women underwent prenatal ultrasound examination or magnetic resonance imaging performed by experienced maternal fetal medicine specialists and ultrasound technicians. The pregnant women ranged in age from 21 to 43 years, with their gestational week ranging between 13 to 32 weeks. The eligibility conditions for inclusion in this study were isolated CFMs and non-isolated CFMs (with other structural abnormalities or sonographic soft markers). According to the International Society of Ultrasound in Obstetrics and Gynecology (ISUOG) guidelines [27] and the recent detailed ultrasonographic report of prenatal CFMs [28], the CFMs included cranial malformations (such as abnormal size, shape, and integrity of the skull) and various facial structure abnormalities in coronal, transverse, and sagittal planes (such as abnormal number, size, shape, mass, or location of orbits, lens, palpebral fissure, mandible, maxilla, forehead, nose/nostrils, lips, tongue, soft palate, or ears). Of note, nasal bone absence or dysplasia as sonographic soft markers were excluded from the facial malformations. Fetal samples were obtained from aborted fetuses (14 cases) or were collected by amniocentesis (40 cases) or cordocentesis (64 cases) based on the gestational week at the time of invasive prenatal testing.

### Karyotype analysis

A total of 104 fetal samples (14 fetal tissues from abortions were excluded) were analyzed using standard G-banded karyotyping at 320–450 bands resolution to diagnose overall chromosomal abnormalities. At least 20 metaphase cells from each sample were carefully examined by an experienced technician to detect chromosomal structural abnormalities and numerical abnormalities.

### Chromosomal microarray analysis

CMA was performed on all samples from the 118 cases using the Illumina Human CytoSNP-12 Array or the Affymetrix CytoScan 750 k Array, according to the respective manufacturers’ instructions. The results were analyzed with Chromosome Analysis Suite software. All detected CNVs were compared with known CNVs in the scientific literature and in the following publicly available databases: DECIPHER (http://decipher.sanger.ac.uk/), Database of Genomic Variants (DGV, http://dgv.tcag.ca/dgv/app/home), Online Mendelian Inheritance in Man (OMIM, http://www.omim.org), ClinGen Dosage Sensitivity Map (https://www.ncbi.nlm.nih.gov/projects/dbvar/clingen), and International Standards for Cytogenomic Arrays (ISCA, https://www.iscaconsortium.org/).

According to the American College of Medical Genetics Standards and Guidelines, the CNVs were classified as pathogenic, likely pathogenic, benign (B), likely benign (LB), or variant of unknown significance (VOUS) [29, 30]. Given the reliability of the minimum detection range, the reporting threshold for P or LP CNVs was 100 Kb; LB/B CNVs and VOUS CNVs smaller than 500 Kb deletion or 1000 Kb duplication were not reported, consistent with the Canadian College of Medical Geneticists (CCMG)-Society of Obstetricians and Gynaecologists of Canada (SOGC) recommendations [31]. All reported CNVs were according to the National Center for Biotechnology Information human genome build 37 (hg 19). CMA or quantitative real-time polymerase chain reaction was also performed on parental DNA samples, if DNA were available, to determine whether the CNVs detected were inherited or de novo.

To identify CFM-associated CNVs and candidate genes, we further examined and analyzed the genes within identified CNVs by retrieving related literature and examining related databases.

### Statistical analysis

Statistical analysis was performed with SPSS 23.0 (IBM Corporation, USA). The CMA detection rates of P/LP variants were compared between various isolated and non-isolated CFM fetuses, and simple and complex CFM fetuses; *p* < 0.05 was considered statistically significant.

## Data Availability

The data that support the findings of this study are available from the corresponding author upon reasonable request.

## Abbreviations

CMA: Chromosomal microarray analysis
CFM: Craniofacial malformation
CNV: Copy number variation
P: Pathogenic
LP: Likely pathogenic
LB: Likely benign
B: Benign
VOUS: Variant of unknown significance
MMS: Microdeletion or microduplication syndrome
